# An atypical facial eruption in skin of color: A rare presentation of histiocytoid Sweet syndrome

**DOI:** 10.1016/j.jdcr.2024.02.027

**Published:** 2024-03-09

**Authors:** Brittany Ehlert, Lauren Shegos, Matthew Franklin, Kaila Buckley, Jaimie Rodger

**Affiliations:** a Ohio University Heritage College of Osteopathic Medicine, Cleveland, Ohio; b Department of Dermatology, OhioHealth Riverside Methodist Hospital, Columbus, Ohio; c Department of Dermatology, Oakview Dermatology, Gahanna, Ohio; d Department of Pathology, OhioHealth, Columbus, Ohio; e Department of Dermatology, Epiphany Dermatology, Greenville, South Carolina

**Keywords:** acute febrile neutrophilic dermatosis, histiocytoid Sweet syndrome, Sweet syndrome

## Introduction

Sweet syndrome (SS), or acute febrile neutrophilic dermatosis, was first described by Robert Sweet in 1964 and is characterized as tender, erythematous papules, or nodules that histologically consist of mature neutrophils with edema of the dermis.^1^ These lesions are commonly located on the upper extremities, trunk, head, and neck and are accompanied by fever, neutrophilia, and leukocytosis. The 3 subtypes of SS include the following: (1) classical neutrophilic Sweet syndrome, which is associated with upper respiratory or gastrointestinal infections, pregnancy, or inflammatory bowel disease; (2) malignancy-associated Sweet syndrome, which can be seen in patients with undiagnosed or established cancer; and (3) drug-induced Sweet syndrome, which often occurs in patients treated with granulocyte colony–stimulating factor.^1^

In 2005, Requena et al^2^ described histiocytoid Sweet syndrome (HSS) as a histological variant of SS that demonstrated papillary dermal edema with lymphocytes and immature myeloid cells that resembled histiocytes rather than the neutrophils seen in SS.^2^ CD-68 and myeloperoxidase (MPO) stains were used to confirm the cells seen on histology were in fact neutrophil precursors and not true histiocytes.^3^

The gold standard treatment for SS is corticosteroids, but potassium iodide and colchicine have also been considered as first-line therapy.^1^ Second-line treatment includes clofazimine, cyclosporin, dapsone, and indomethacin. Although these agents are considered second-line therapy, they have been used for both refractory cases and as initial therapy.^1^

## Case report

**Fig 1:**
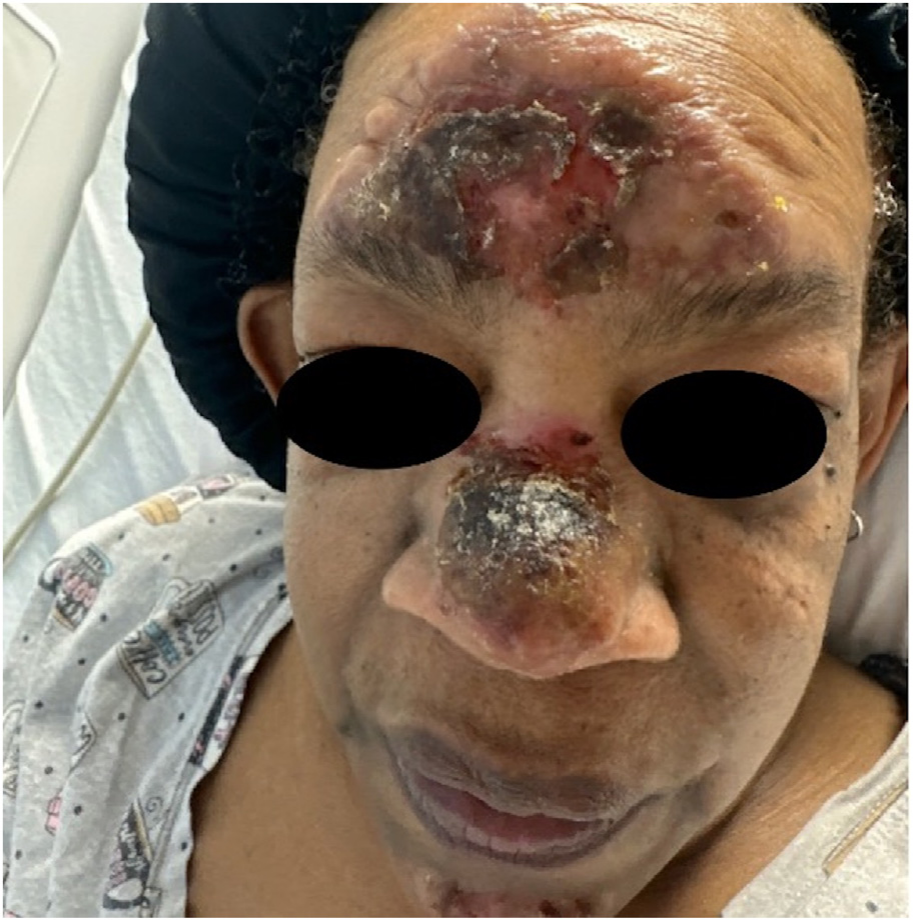
Initial presentation of violaceous, edematous vegetative, and ulcerated plaques involving the forehead, nose, and chin with sparing of the nasal bridge.

**Fig 2:**
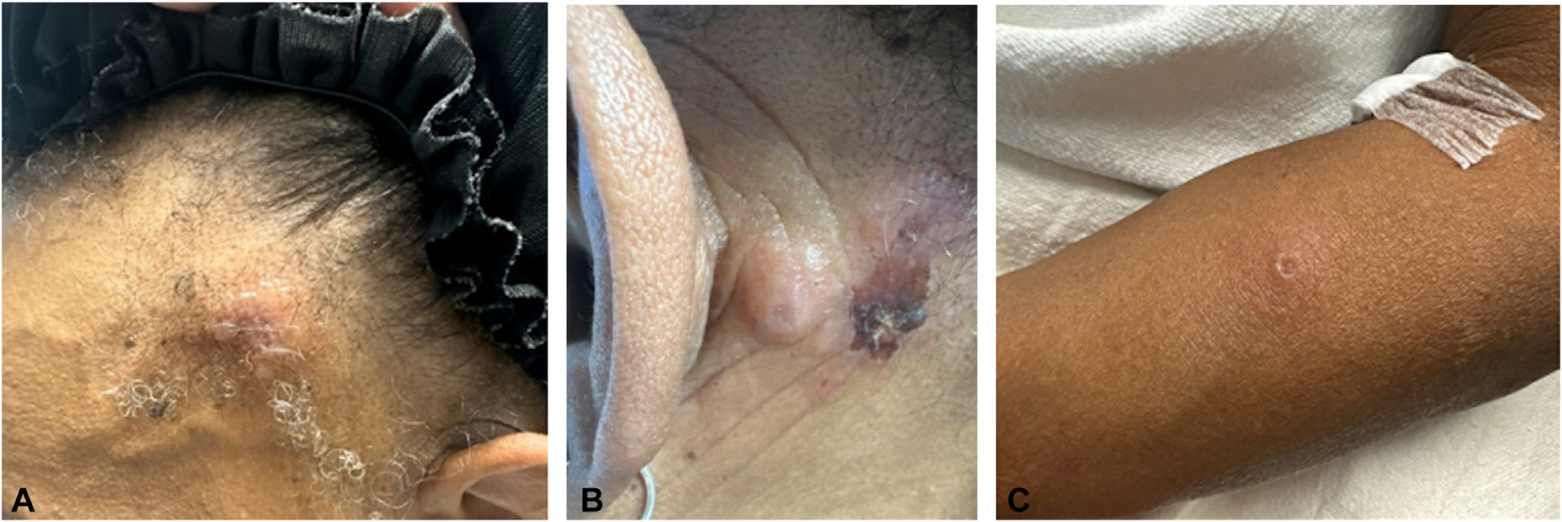
Initial presentation of erythematous smooth umbilicated papules and nodules involving the (**A**) left side of the frontal aspect of the scalp, (**B**) left auricular neck, and (**C**) left arm.

A 64-year-old woman with a past medical history of diabetes, hypertension, and hypothyroidism presented with a 3-week history of violaceous, edematous, vegetative, and ulcerated plaques involving the forehead, nose, and chin with sparing of the nasal bridge (Fig 1). Additionally, she exhibited few, erythematous, smooth, umbilicated papules, and nodules involving the left arm (Fig 2, *A-C*). The rash was refractory and progressive to previous oral and topical antibiotics. She endorsed associated facial pain and a mild cough, but denied other symptoms, new medications, recent travel, or excess ultraviolent radiation exposure. She was afebrile on presentation.

Given the broad infectious and inflammatory differential diagnoses, an extensive workup was performed. Laboratory testing and imaging revealed elevated inflammatory markers but was otherwise unremarkable including negative infectious and autoimmune workups. Serologic titers for antidesmoglein antibodies were not detected. Biopsy for tissue culture was negative and the initial biopsy for pathology was consistent with ulcerated skin with mixed dermal inflammation. Repeat biopsy for pathology revealed marked papillary dermal edema with superficial and deep perivascular and interstitial lymphohistiocytic inflammation with negative immunohistochemical staining for MPO and Ebstein-Barr virus-encoded RNA.

**Fig 3:**
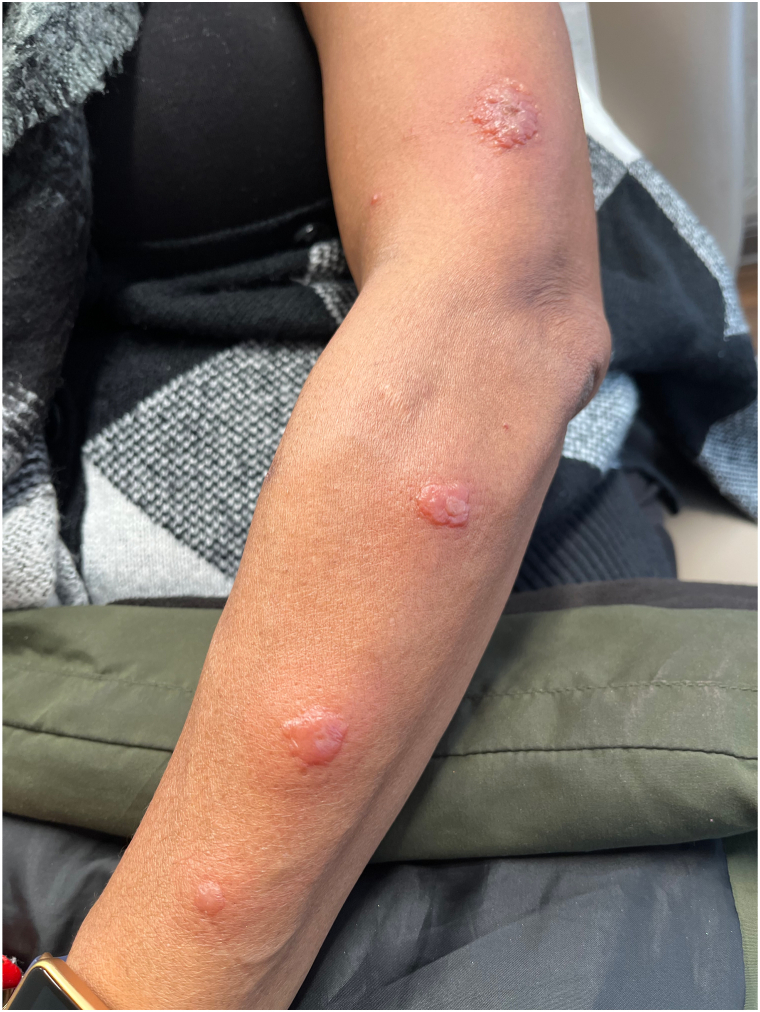
Flare of umbilicated nodules after completing course of prednisone.

Correlating the clinical presentation of edematous facial plaques with the histologic findings of marked papillary dermal edema with perivascular lymphohistiocytic inflammation, our working diagnosis included atypical polymorphous light eruption versus discoid lupus. However, upon completion of a 21-day systemic corticosteroid taper, the patient represented with fevers, malaise, and recrudescence of lesions (Fig 3). An additional biopsy was performed, which revealed a negative direct immunofluorescence and mild epidermal acanthosis and spongiosis with prominent papillary dermal edema and dense stromal inflammation with now strongly positive MPO staining suggestive of HSS.

Upon diagnosis, referral to oncology for malignancy workup was performed and unrevealing. She was started on low dose prednisone and transitioned to oral dapsone maintaining clearance of skin lesions.

## Discussion

Although SS does not have racial predilection, the clinical diagnosis often relies on identification of the classic erythematous to violaceous lesions, which as with our case, can be more challenging to identify in skin of color.^1^ SS typically occurs on the head, neck, upper extremities, or trunk and is associated with constitutional symptoms such as fever^2^; however, our patient was initially afebrile with predominately facial lesions. Additionally, the atypical presentation of vegetative plaques (Fig 1) led to a very broad clinical differential.

The infectious workup was pan-culture negative with negative infectious stains, including Grocott methenamine silver, Giemsa, acid-fast bacillus, Fite, and herpes simplex virus. The inflammatory workup was also negative for antineutrophilic cytoplasmic antibody negative and granulomas. Biopsy results showed prominent papillary dermal edema and dense stromal inflammation with strongly positive MPO staining and negative Epstein-Barr virus–encoded RNA, which eliminated the remaining differential diagnoses such as hydroa vacciniforme-like lymphoproliferative disorder and supported a diagnosis of HSS.

Diagnostic criteria for SS include the presence of both major criteria: (1) abrupt onset of typical cutaneous lesion and (2) histopathology consistent with SS, and at least 2 minor criteria: (1) preceded by 1 of the associated infections or vaccinations, accompanied by 1 of the associated malignancies or inflammatory disorders, or associated with drug exposure or pregnancy; (2) presence of fever and constitutional signs and symptoms; (3) leukocytosis; and (4) excellent response to systemic corticosteroids. Our patient did meet major criteria for SS as well as the second criterion due to her fever and malaise and excellent response to systemic steroids. However, there is not diagnostic criterion established for HSS, rather it is based on a histologic diagnosis.^2^

The gold standard of diagnosis for HSS is biopsy with confirmatory MPO staining. Histologically, SS characteristically has an infiltrate of mature neutrophils in the dermis.^2^ HSS, a histological variant of SS, instead demonstrates immature neutrophils that resemble histiocytes. To differentiate between immature myeloid cells and histiocytes, MPO staining is used.^2^ It is critical to correctly diagnose HSS due to its association with malignancies.

Multiple cases of patients with myelodysplastic syndromes (MDS) being diagnosed with HSS have been reported. Shalaby et al^4^ reviewed 7 cases of HSS in MDS patients ranging from 44 to 75 years of age (average age of 65 years) and noted 4 out of the 7 patients were women. Another study done by Ghoufi et al^5^ compared frequency and type of hematological malignancies associated with HSS versus classical neutrophilic SS. They reported that hematological malignancies and MDS were more frequently associated with HSS, and lymphoid malignancies were less frequently associated with HSS compared with classical neutrophilic SS.^5^ Although majority of patients in the study were diagnosed with hematologic disease before their HSS diagnosis, there were 3 patients diagnosed with MDS diagnosis within 6 months after their HSS diagnosis. For this reason, this study concluded that a complete hematologic investigation is imperative with a HSS diagnosis and recommended continued screening until at least 6 months after initial diagnosis to promptly diagnose a hematologic malignancy.^5^ Our patient had a negative MDS work-up, but we will continue to screen for MDS and malignancy in the future.

Corticosteroids are considered first-line therapy for HSS, and our patient was initially treated with corticosteroids with temporary relief. Alternative therapies have been used as initial treatment or after treatment failure and include potassium iodide, colchicine, indomethacin, dapsone, clofazimine, and cyclosporin.^1^ After failure with corticosteroid treatment, our patient was successfully treated with dapsone as monotherapy, which is typically considered second-line therapy.

## Conflicts of interest

None disclosed.
